# The Treatment of Dead Teeth

**Published:** 1891-04

**Authors:** E. Balding


ARTICLE III.

### THE TREATMENT OF DEAD TEETH.

BY E. BALDING.

A paper read before the Students' Society, London Dental Hospital.

*Mr. President and Gentlemen:*

If any apology were needed for choosing such a well-worn subject as that of "Dead Teeth" for our discussion to-night, I offer as such my reasons for doing so, viz., the great number of such teeth we are called upon to treat, and the immense, and to the private practitioner, the vital importance of their successful treatment.

Dead teeth naturally fall into two classes, viz.:— I. Those whose pulps have been devitalized by the application of arsenious acid; and—II. Those which have lost their vitality previous to coming under our care.

I. The teeth of the former class need not detain us long. Here we have to deal with a tooth, the pulp canal or canals of which can never be in a more aseptic condition, and provided the pulp is withdrawn successfully, and the canals properly filled, there is not the least doubt as to the result of our work. There is no class of teeth more favorable for immediate root filling than this, and almost invariably this is the best treatment to adopt.

The first step is to thoroughly open up the pulp-chamber so as to get direct access to the canals. In the case of interstitial cavities, this sometimes involves the cutting away of much of the crown of the tooth, and is easily effected with one of the new cross-cut burrs. Some operators advise, instead of extending these cavities, to make an entirely fresh opening by drilling straight through the crown into the pulp chamber, but there is very little advantage gained by this method, unless the bridge between the two entrances to the cavity is tolerably strong. Next, an attempt should be made to withdraw the pulp whole. This may be done by passing a very fine flexible watchmaker's brooch right to the extremity of the canal, and giving it one or two turns in order to sever its connection at the apex. Very often on withdrawing the brooch, the pulp will come away with it, but if it does not, it may be secured by passing up a barbed Donaldson bristle, or other nerve extractor. Should this also be unsuccessful, use with the engine a very fine Gates-Glidden drill which has had the point of the blade broken off, and this seldom fails to entangle the pulp and bring it away.

After cutting away all overhanging dentine from the canals, so as to make their orifices funnel-shaped, mop them out with absolute alcohol, or better still, a one per cent. solution of perchloride of mercury in absolute alcohol, and dry with the hot air syringe, or by inserting a piece of wire, made hot in the flame of a spirit lamp. There is no object. neither any advantage gained, but considerable risk run by enlarging the canal with a drill, except perhaps where the contents are partly calcified towards the proximal end. Having previously gauged the length of the canal by marking it on a brooch with a small disc of rubber dam, and the root being thoroughly cleansed and dried, we proceed to introduce the permanent root filling, and over this the permanent plug.

And this is simple enough, some one says, but what would you do in a case where the canals are so small as not to admit the passage of even a very fine bristle, as is commonly the case in the buccal roots of upper molars, and the anterior roots of lower molars? To this I reply that I should leave them alone. Of course the proximal end of such a canal should be somewhat enlarged to make sure that it is not a deposition of secondary dentine which is merely blocking the opening, and if it is not so, the canal may very safely be left with some antiseptic in the enlarged part. Some little time ago exactly such a case came under my notice. The patient had a carious six year upper molar, and on examination I discovered a slight exposure, to which I applied arsenious acid. The patient came again in two days, and on opening up the pulp chamber I removed the pulp from the palatine canal without any difficulty, but on looking for the buccal canals, I could only make out two very minute openings which would not admit more than the point of the finest brooch. I endeavored to enlarge them, first with a Hern burr, and then with a drill, but as the obstruction continued I did not persist, but mopped out the cavity with perchloride of mercury, and inserted gutta percha points into the palatine canal, filled the pulp chamber with osteo and put in a gold plug. I saw this patient two days ago, and found that the tooth had not given the slightest uneasiness since it was filled. With these few words we will pass over the teeth of the first class.

II. The teeth of the second class—those which have lost their vitality previous to coming under our care—may be grouped into three subdivisions, which we will consider *seriatim*.

(*a*.) Under the first may be included those of which there is no history of alveolar abscess, but whose pulp has died either as the result of accident or disease. When this is the case, the contents of the canal will generally be in a state of putrid decomposition, and if the pulp chamber has been encroached upon by caries or otherwise, may be pouring out pus into the cavity. Sometimes when the pulp has died spontaneously without any obvious cause, it will be in a semi-liquefied condition, known as moist gangrene, or less often we may find it shrivelled up as in dry gangrene, and more rarely still it may seem to have disappeared altogether. But whatever may be the pathological state of such teeth, their actual condition is by no means as favorable as that of the class we have just considered, and in their treatment it is impossible to exercise too much care. Proceeding as before, we remove all loose debris, and clear out the canal as well as possible, working all the time in the presence of, aad in the case of lower teeth, under a pool of some antiseptic, as creosote or peroxide of hydrogen, or a 1-250 solution of perchloride of mercury. The walls of the canal will probably be softened and soaked with the putrid contents, and now the Gates drill is called into requisition; it should be fine and used with a "touch-and-go" movement, continuing the use of the antiseptic, and with our gauge set on the drill, there should not be much fear of forcing any debris through the apical foramen. Now the canal must be thoroughly dried, and this is best done by mopping out with absolute alcohol, and then using the hot-air syringe, the alcohol having the power of dehydrating the dentinal tubes, and so causing them the more readily to take up whatever antiseptic may be used. There are now two courses of treatment open to us, in the adoption of either of which we must be guided by a careful consideration of the circumstances of each individual case. If there has not been the slightest indication of periostitis, or any pain whatever, and there is no reason to expect an untoward result from any other cause, such as absorption at the root, etc., we might feel perfectly justified in permanently filling the root and tooth at once. But should the conditions not prove so favorable, or should any doubt exist in our mind as to the result of such treatment, there can be nothing lost by delaying the permanent operation, and inserting a dressing of eucalyptus oil and iodoform. This is I think preferable to carbolic and iodoform, because the eucalyptus dissolves the iodoform, and being very volatile diffuses itself into every space left unoccupied.

The cavity is now filled with a plug of temporary gutta percha, and if at the end of a week the tooth has remained quite comfortable, the dressing may be exchanged for a permanent root filling, and a plug of gold, or whatever filling be most suitable, inserted. In cases where the patient cannot be seen again, and there has been slight periodontal irritation, caused no doubt by the presence of septic substance, Mr. Coleman recommends the following treatment:—Clear out the pulp cavity, wash out with carbolic acid, and lay over the opening a small disc of card saturated with carbolic, and having attached to the surface looking into the canal a small quantity of arsenious acid (about 1-16 grain). This is covered over with oxychloride of zinc, and the remainder of the tooth filled with gold or amalgam.

(*b.*) The second subdivision will include those teeth at whose root a blind abscess exists, or is in process of formation. Here our treatment may vary according to the stage to which the abscess has advanced. If it seems just beneath the gum, or if its actual position can be made out at all clearly, the treatment will be much simplified by anticipating nature by cutting down on or drilling into the sac from the gum over it, and treating as an abscess with a fistulous opening. If the abscess has not so far advanced as to warrant this course of treatment being followed, then the canal should be cleared as thoroughly as possible, and a loose cotton wool dressing with a little iodoform or perchloride of mercury inserted, and changed every day until it comes away clean and sweet. Some operators advise going through the apical foramen with a very fine drill so as to tear up the sac and draw the blood, and then putting in a dressing as soon as all bleeding or weeping into the canal has ceased. Although my experience has not been very great, I have found very good results follow this latter course of treatment in the few cases in which I have tried it; but whichever course is adopted, I should not advocate the practicing of immediate root filling.

Sometimes, however, by the force of circumstances we may be driven to adopt some immediate treatment, and the only one that occurs to me besides that of filling the root and treating the abscess externally, is that known as rhizodontrophy, which consists in capping over the pulp cavity, and at once filling the tooth; after which a fine hole is drilled either through the side of the tooth, just below and through the margin of the gum or through the filling itself into the pulp cavity. But in any case, as an aid to our treatment, the gum over the affected tooth should be dried and strongly and repeatedly painted with a mixture of equal parts of the double tincture of iodine and Fleming's tincture of aconite; in fact I deem it advisable to do this as a preventive after treating any tooth where there is any likelihood of inflammation being set up or increased.

(*c*.) The third and last subdivision comprises those teeth which are the subjects of an alveolar abscess, which having burst, has left a suppurating tract, opening generally at the summit of a pustule which is sometimes 1-8 or 2-16 of an inch long. Every now and again a small gum-boil makes its appearance, enlarges, bursts and subsides for a while, only to reappear again and again. As a rule the patient experiences no pain from it, neither any inconvenience beyond that caused by the occasional liberation of the very offensive pus into the mouth. The majority of these cases are amenable to immediate treatment, which consists briefly in getting a few drops of some powerful antiseptic to traverse the whole length of the pulp canal and the sinus leading from its apex, and then permanently filling both the root and the crown. To my mind, the simplest and most satisfactory method of doing this is as follows:—Clear the canal as well as possible, and then cautiously but thoroughly cut away the immediate wall with a Gates drill; pass a very fine brooch up the canal so as just to force its point through the apical foramen, and then dry the canal. Now take the needle of a hypodermic syringe, and pass it as far up the canal as possible, without using force; pack tightly all round it with gutta-percha, and having filled the syringe with the antiseptic, screw it on to the nozzle which carries the needle, then with very little pressure we shall almost immediately see our antiseptic oozing out at the sinus on to the gum. The best antiseptic for this purpose is strong carbolic acid, because one can tell by the white eschar it makes when it has reached the gum, and also because being such a strong escharotic, it cauterizes the coats of the sinus, and quickly causes it to heal up by granulation. Some prefer to use a less potent drug, as creosote, or peroxide of hydrogen, but when these are used it is necessary to allow a moderately good stream to pass through the channel before proceeding with the operation, and even then the cure is neither so sure nor so quick. In the case of carbolic it is only necessary to let one drop appear on the gum to effect a cure. When this has been done, remove the syringe and the surrounding gutta-percha, dehydrate the canal with absolute alcohol, mop out with perchloride of mercury, dry again, and insert the permanent filling both in the root and the crown. If a hypodermic syringe is not at hand, a tapering piston may be made to fit the canal, by winding cotton wool tightly round a brooch, dipping freely in carbolic and using with a pumping action till the white mark appears on the gum. About six weeks ago I inserted a gold filling in an upper central that had an abscess at its root with a fistulous opening, having previously treated it in this way and filled the roots immediately with gutta-percha points, the result being entirely satisfactory. If the abscess is one of very long standing, a variable amount of absorption may have taken place at the root. Sometimes on extracting a tooth that has refused to yield to any other kind of treatment, we find that perhaps 1-16 of an inch has disappeared from the apex in this way, and we shall be pretty safe in suspecting this to be the case in almost any tooth of this class, which does not seem disposed to yield to our conservative treatment, and then our only alternative is to attempt the radical cure.

About a year ago a case of some interest came under my notice. The patient, a man of about thirty, had an abscess in the lower jaw, and a sinus leading from it opening under the chin close to the symphysis. After enduring an intermittent discharge from it for three years, he came to this hospital and on examination it was found that the trouble proceeded from the right lower central, which was dead, but without the slightest sign of caries. An opening was made into the pulp chamber, and an attempt made to force some peroxide of hydrogen down the canal out on the chin, but without success, although there was considerable enlargement of the apical foramen, so a temporary dressing was inserted, and the patient seen every other day, the dressing being renewed each time. The discharge gradually lessened, and when it ceased the tooth was filled permanently. Since then there has been no further recurrence of the trouble, and the tooth which at first was loose has grown quite firm.

On the subject of root fillings a large field is opened to us for discussion, for the selection of a suitable filling is as important as it is an essential item in the treatment of dead teeth. Our one aim in filling a root is to effectually and permanently close the apical foramen, and so prevent the canal from becoming the receptacle of any bloody, serous or purulent exudation, therefore in stating the requirements of a good root filling I should place them in this order:—1st. That it should go the extremity, and this almost always necessitates its being solid. 2d. It must be easy of introduction, and adaptation to the various conditions of shape and size met with in different canals; 3rd. It must be capable of being easily removed, should circumstances require. One writer, I believe Mr. Tomes, also adds that it should be antiseptic. Therefore a perfect root-filling may be defined as a solid substance that will penetrate to the apex of the canal, is antiseptic, non-irritating and readily removable. The number of root-fillings in use are practically innumerable, almost every operator having his own particular favorite, and thinking it in his heart to be the only right one, and if it were possible to enumerate them all, I would not take up your time by doing so, but still a brief allusion to some of those in ordinary use might not be considered out of place here. From being conservative in our treatment of teeth, I think we too often allow ourselves to get conservative in the use of root-fillings. In the selection of our material, we should be guided by a judicious consideration of the circumstances and conditions of each individual case, and choose our filling accordingly.

Of all root-fillings I have used up to the present, the most successful and the one answering most nearly to all the necessary requirements is gutta-percha in the form of fine tapering points. The method of using these is briefly as follows:—We will suppose that the canal has been cleared out, disinfected and thoroughly dried, as it should be before the introduction of any filling, it is now freely mopped out with chloroform, or a solution of gutta percha in chloroform, and taking one of these points in the conveying forceps, dip for an instant into chloroform and plunge to the end of the canal. It is best to cut the points to the length of the canal previously by the use of our gauge. If there is a plenty of chloroform in the canal it is not necessary to dip the points in it, as then there is the greater chance of their going home. If the canal is large it will take two or perhaps more of the points, and when full allow two or three minutes for the chloroform to evaporate, and then condense with a nerve canal plugger, and proceed to insert the permanent plug with or without a floor of osteo.

Next, in order of merit, I should be inclined to put a gold or copper wire in conjunction with chloropercha. The wire is drawn out very fine, and the end made slightly tapering. The canal is freely wiped out with chloropercha and the wire inserted, having itself been previously dipped in the solution. Some consider this superior to the gutta percha, as it is stiffer and more likely to go to the extremity, but like the gutta percha, and, in fact, all other "points," it should be cut to the length of the canal as measured by our gauge.

Wood points, soaked in carbolized paraffin, may also be used in the same way, or in conjunction with oxychloride of zinc.

Shellac, mixed with a small quantity of wax, makes a very good filling. It is drawn out into extremely fine threads, with which the canal is filled, and the whole flooded with alcohol; as soon as they begin to soften more points are inserted till the canal is full. Iodoform wax on cotton wool is a filling that is used a great deal, and but for the difficulty, and even impossibility, to get it to go to the extremity in small canals it might be called a perfect filling. You are all familiar with the way in which it is used, so I will not take up your time by describing it, but I think the remarkable length of time it preserves its antiseptic power is worthy of note.

Another filling consists of paraffin placed in the orifice of the canal and melted in by passing hot bristles down. I have never had any experience of this mode of filling, but should think that it is only practicable in teeth in the lower jaw.

The oxyphosphate or oxychloride of zinc carried up on shreds of cotton wool with a bristle, is much recommended by some operators, but the great objection that it has is the difficulty of working, combined with the almost impossibility of getting it to the extremity, and when once up getting it down again; of course it is needless to add that it should be mixed very thin. Some discountenance the use of the oxychloride as being liable to set up irritation through the apex, while others prefer it because of its antiseptic properties.

Gold and tin foil being quite out of date and unsatisfactory in their use for root filling, I do not think it necessary to dwell upon them to-night.

A very fair filling is made of plaster of Paris, mixed thin with a one per cent. solution of perchloride of mercury. The canal is first wiped out with the perchloride and then the plaster coaxed up with a bristle or on shreds of cotton wool, but no time must be lost in this operation or the plaster will set before the canal is properly filled, and some minutes should be allowed for the plaster to harden before the permanent plug is introduced.

Amalgam is another filling that is patronized by a goodly number of operators, the only fault I have to find with it is, that once in it is a fixture and is practically impossible to get out. Sullivan's amalgam is the best to use for this purpose on account of its preservative, antiseptic, and hardening action; but before inserting, the apex of the canal should be closed with a scrap of gutta percha.

A mixture of tin and lead, made into points like the gutta percha, has been used sometimes, but not having had any experience of them I cannot say how they answer.

During the last year Mr. Tomes has introduced a new root-filling in the form of celluloid, such as is used to carry the sensitive photographic film. The *mode d'emploi* is as follows:—The celluloid is first cut into exceedingly fine strips, the canal having been thoroughly dried is mopped out with collodion, and one of the strips is taken in the plugging forceps, dipped into the collodion, and coaxed up the root, after which guncotton is carried up on bristles and packed in. This is very similar to the gutta percha points, with the exception that the celluloid is square at the end while the gutta percha is pointed, an exception which I think goes in favor of the gutta percha. The advantages claimed for this by Mr. Tomes, are that the strips so prepared have considerable stiffness, and are yet flexible and very tough, and also that they make an antiseptic and absolutely non-irritant filling.

Lastly, we have a number of wool dressings, which, although they make admirable dressings, are not admissible as permanent fillings, albeit they are sometimes used as such. These chiefly consist of iodoform and eucalyptus oil, carbolic, creosote, perchloride of mercury, and arsenic.

Iodoform and eucalyptus has been known to preserve its smell after being in a tooth several years, but carbolic and creosote lose their power after a short time. The use of arsenic has been recommended by Mr. Coleman in certain cases because of its very powerful property.

In conclusion, allow me briefly to recapitulate those points of treatment which are of prime importance. First, there is the free exposure of and direct access into the pulp chamber and the openings of the canals. Then the thorough cleansing, disinfecting, and dehydration of the roots, and finally their accurate and complete filling.

It only remains for me to thank you, gentlemen, for the patience with which you have listened to my desultory paper. I feel that I have done but scant justice to a subject on which volumes have been written by far abler pens than mine, but which is still the vortex of a whirl of interminable debating. I can only hope that what is deficient may be supplied by a lively discussion.—*Dental Record.*

